# Defect spectroscopy on the dielectric material aluminum oxide

**DOI:** 10.1038/s41598-020-69240-3

**Published:** 2020-07-27

**Authors:** Dennis Oing, Martin Geller, Lucas Stahl, Jens Kerski, Axel Lorke, Nicolas Wöhrl

**Affiliations:** Department of Physics and CENIDE, University Duisburg-Essen, Lotharstr. 1, 47057 Duisburg, Germany

**Keywords:** Materials science, Physics

## Abstract

A method for defect characterization is presented that allows to measure the activation energy, capture cross-section, and defect density in dielectric materials. This is exemplarily performed on aluminum oxide thin films deposited on hydrogen-terminated diamond. During the measurement, samples were illuminated using a 405 nm laser, charging the defects while simultaneously measuring the surface conductivity of the diamond at different temperatures. By applying the standard boxcar evaluation known from deep-level transient spectroscopy, we found five different defect levels in $\hbox {Al}_2\hbox {O}_3$. One can be identified as substitutional silicon in aluminum oxide, while the others are most likely connected to either aluminum interstitials or carbon and nitrogen impurities.

## Introduction

Defect characterization is important for material science as defects influence in particular the optical and electrical properties of materials^1, 2^. Hence, a variety of characterization methods have been developed. These methods include optical techniques, e.g. photoluminescence spectroscopy^3^, and electrical techniques, such as deep-level transient spectroscopy (DLTS)^4–6^, as well as optoelectrical techniques, e.g. photocurrent spectroscopy^7, 8^. Each method has its advantages and disadvantages. Therefore, the different methods complement each other as there is not a single method to fully characterize any material.

A material class that is routinely characterized using these methods are semiconductors. The position, concentration and energy level of defects in semiconductors are easily studied by deep-level transient spectroscopy^4^. Since its first appearance, this powerful method has found many applications, e.g. deep-level optical spectroscopy^9^ and time-resolved capacitance spectroscopy on semiconductor nanostructures^10–12^.

However, the standard DLTS technique uses a depletion capacitance of a p-n or Schottky junction in a doped semiconductor to obtain the activation energies and capture cross section of the defect states. Hence, dielectric materials cannot be characterized using conventional time-resolved capacitance spectroscopy. Defect states in dielectic materials, for instance $\hbox {Al}_2\hbox {O}_3$, have been studied by electrical charge injection into the insulator in a charge-trapping non-volatile memory structure^13, 14^. However, only the defect density and their position from the interface have be studied, leaving their activation energies and capture cross sections unknown.

Here, we describe a new optoelectrical technique, which uses the boxcar-evaluation method from DLTS that enables the characterization of defect levels in an undoped dielectric material. In this technique, a two-dimensional charge carrier gas at the interface between two dielectric materials is utilized as sensor for the charging and discharging of the defect levels. It has already been shown that the conductance of a two-dimensional charge carrier gas can be used to probe charging and discharging events with a higher sensitivity than capacitance measurements^15^. In the present study, charging of the defect levels is performed using optical excitation, while the temperature-assisted discharging is monitored electrically, using the two-dimensional carrier gas.

The method is performed here on aluminum oxide ($\hbox {Al}_2\hbox {O}_3$) that was deposited on hydrogen-terminated diamond. The hydrogen termination is known to induce a two-dimensional hole gas (2DHG) on the diamond surface^16^, which is used here for the electrical readout by conductance measurement. The method is not limited to $\hbox {Al}_2\hbox {O}_3$, since the hydrogen-terminated diamond surface can be used for other dielectric materials as well. Also, other two-dimensional carrier gases (e.g. such as oxide-oxide interfaces^17^) or even two-dimensional materials, such as graphene or transition metal dichalcogenides, may be employed in place of hydrogen-terminated diamond.

## Methods

**Figure 1:**
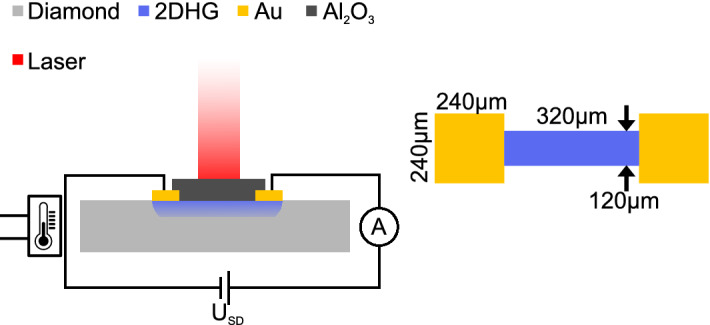
Schematic of the set up used in this study. Heating up to 220 °C is possible.Two different lasers (405 nm and 850 nm wavelength) were used in this study. The geometry of the sample is shown on the right.

The single-crystalline ${<}100{>}$-oriented diamond sample was grown by plasma-enhanced chemical vapor deposition. The deposition process and hydrogen termination of the substrate surface have been reported elsewhere^18^. Ohmic contacts to the 2DHG were fabricated by evaporation of 10 nm Ni and 100 nm Au and by using standard optical lithography. The dimensions of the contacts are shown in Fig. 1. $\hbox {Al}_2\hbox {O}_3$ films were synthesized in a custom-made anodic arc discharge chamber. Aluminum is melted and evaporated by applying a discharge current of 55 A producing a metallic plasma. An oxygen flow is set to 500 sccm during the evaporation resulting in a chamber pressure of $2.5\cdot 10^{-2}\,\hbox {mbar}$. These conditions were found to form an $\hbox {Al}_2\hbox {O}_3$ layer with the right degree of oxidation on the hydrogen-terminated diamond surface. X-ray photoelectron spectroscopy was used to verify the stochiometry of $\hbox {Al}_2\hbox {O}_3$. Two samples (labelled “1” and “2”) with nominally identical growth parameters were investigated in this study.

The 2DHG was characterized on sample 1 by temperature-dependent Hall measurements in the range of $4\,{\hbox {K}}\le T \le 300\,{\hbox {K}}$ before and after $\hbox {Al}_2\hbox {O}_3$ deposition. Additionally, the conductance was measured in four-point geometry to determine the mobility.

The sample was mounted in a vacuum chamber which can be evacuated to a base pressure of $3\cdot 10^{-1}\,\hbox {mbar}$. A vacuum window was installed to illuminate the sample with a laser beam. The laser beam was not focused and has a diameter of 1 mm and covered the whole sample during measurements. The sample was heated using a resistance heater attached to the substrate holder. The heating current was controlled with a proportional-integral controller, which stabilized the temperature to changes of less than 100 mK in 3 h for temperatures ranging between 22 and 220 °C. Hence, the temperature dependence of the properties of the 2DHG are negligible. A schematic representation of the setup is shown in Fig. 1.

A constant voltage $U_{SD}$ was applied between the Ohmic contacts during the measurement and the resulting current in the 2DHG was recorded. We used a violet laser (405 nm) and an infrared laser (850 nm) and illuminated for 1 h to ensure a steady state situation of the 2DHG’s conductance. Afterwards, the laser was turned off for 2 h. The current was measured during the 3 h period with a sampling rate of approximately 3 Hz. This measurement procedure was performed for temperatures between 58 and 211 °C in steps of 1.5 °C and 30 °C up to 180 °C in steps of 4 °C, for sample 1 and 2, respectively.

## Results

Hall measurements revealed a temperature-independent carrier density of $1.4\cdot 10^{13}\,\hbox {cm}^{-2}$ and a mobility of $94\,\hbox {cm}^2/\hbox {Vs}$ at 300 K for the 2DHG before $\hbox {Al}_2\hbox {O}_3$ deposition. After $\hbox {Al}_2\hbox {O}_3$ deposition, the carrier density decreased to $6.7\cdot 10^{12}\,\hbox {cm}^{-2}$, independent of the temperature, while the mobility slightly increased to $100.7\,\hbox {cm}^2/\hbox {Vs}$ at 300 K. These results are in good agreement with values reported in literature for the 2DHG on hydrogen-terminated diamond^18, 19^.

**Figure 2:**
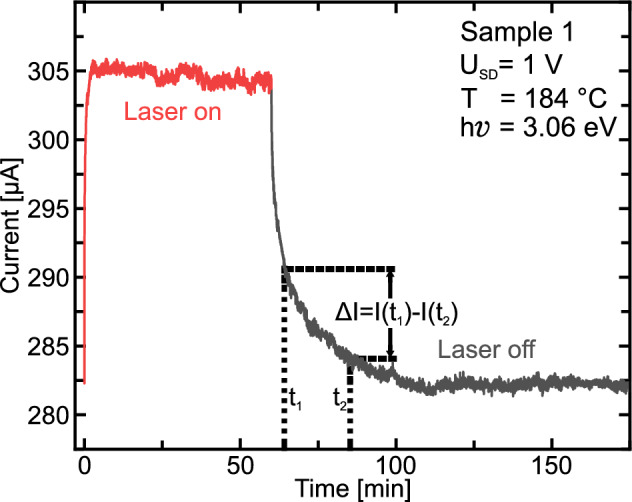
Typical transient measurement performed on sample 1. Time resolved current change measured during illumination of the sample (red part of the curve) and after turning off the laser (gray part of the curve). For the DLTS analysis, the current change $\Delta I$ is evaluated for a given time window between $t_1$ and $t_2$.

As shown in Fig. 2, under illumination with the 405 nm laser, the current increases within a few minutes and reaches a steady state (red line). After turning the laser off, the system relaxes back into its initial state (grey line).

**Figure 3:**
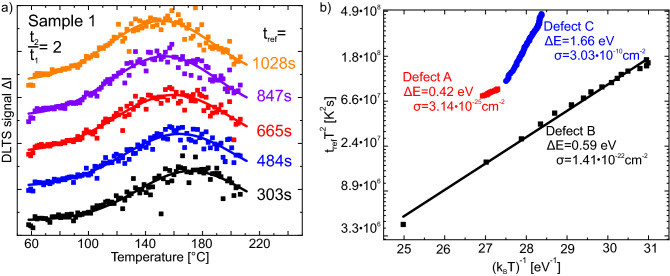
(**a**) Several DLTS spectra (squares) of sample 1 for the investigated temperature range. Full lines show gaussian fits for each spectrum. For this set of data a constant ratio of $t_2/t_1=2$ was chosen. (**b**) Arrhenius plot for sample 1. The temperatures on the x- and y-axis are the temperature position of the maxima of the gaussian fit of the DLTS spectra. Three defects were identified with activation energies of 0.42 eV, 0.59 eV and 1.66 eV.

An evaluation of the transient was performed similar to that for conventional DLTS as described in Lang^4^. Here, we choose a rate window ($t_1$, $t_2$) to calculate the current change $\Delta I=I(t_2)-I(t_1)$ for a reference $t_{ref}=\frac{t_1-t_2}{\ln (t_1/t_2)}$. The current change was then plotted as function of temperature in Fig. 3a. In this study, only one peak can simultaneously be observed in the DLTS spectra. This is attributed to the small temperature range of $153^\circ \mathrm {C}$ and to the rather large differences in rates for different defects. By further increasing $t_{ref}$, the first peak disappears to lower temperatures while a separate peak appears at higher temperature (not shown). These maxima can be distinguished when plotting in an Arrhenius plot (Fig. 3b). Figure 3b is plotted to directly reflect the form of the Equation $\ln (t_{ref}\cdot T^2)=\frac{\Delta E}{k_BT}+\ln \left( \frac{h^3}{16\pi \cdot m^*\cdot k_B^2\cdot g\cdot \sigma } \right)$ from Muret et al.^20^. Therefore, the activation energy $\Delta E$ can be taken from the slope of a linear fit and the capture cross-section $\sigma$ can be calculated from the interception with the y-axis^4, 20^. To calculate the capture cross-section, we assumed an effective mass $m^*=m_e$ and a degeneracy of the defect of $g=1$. All results for the defects found for both samples are summarized in Table 1.

**Table 1:** Summary of all defects, their respective energies, capture cross-sections and densities found in this study.

Sample	Defect	$\Delta E=E_C-E_T$ (eV)	$\sigma \,(\hbox {cm}^{-2})$	Density $(\hbox {cm}^{-2})$	Possible origin of defect
1	A	0.42 ± 0.08	$3.1\times 10^{-25}$	$8\times 10^{10}$	$\hbox {Al}_{\mathrm{i}}^{3+}$
	B	0.59 ± 0.07	$1.4\times 10^{-22}$	$3\times 10^{11}$	$\hbox {Al}_{\mathrm{i}}^{3+}$, N, C
	C	1.66 ± 0.05	$3.0\times 10^{-10}$	$2\times 10^{11}$	Si
2	D	0.52 ± 0.02	$2.0\times 10^{-24}$		$\hbox {Al}_{\mathrm{i}}^{3+}$
	E	1.06 ± 0.05	$8.0\times 10^{-15}$		N, C

## Discussion

To investigate, whether the time-dependent signal may be associated with defects in diamond, we illuminated a diamond sample prior to $\hbox {Al}_2\hbox {O}_3$ coverage. No change in conductivity of the 2DHG during illumination was observed. However, after evaporation of $\hbox {Al}_2\hbox {O}_3$ the typical exponential change in conductivity of the 2DHG (see Fig. 2), as previously discussed, appeared. Therefore, the found defects are attributed to the $\hbox {Al}_2\hbox {O}_3$ layer on diamond.

In the following, we will propose a mechanism to explain the charge carrier excitation and relaxation processes between the valence band of the 2DHG, the defect states and the conduction band in the $\hbox {Al}_2\hbox {O}_3$.

**Figure 4:**
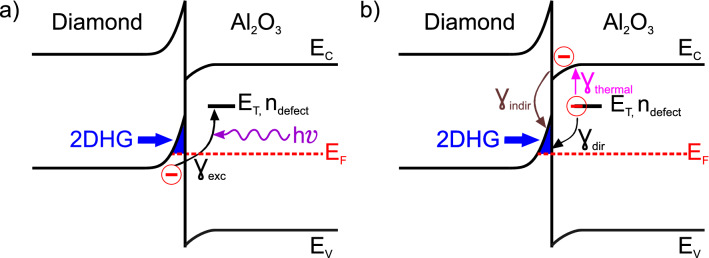
Schematic of the excitation and relaxation processes. (**a**) Electrons from the valence band of diamond are optically excited with a rate $\gamma _{exc}$ into the defect level. The holes that are created this way increase the conductivity in the 2DHG. (**b**) Two possible relaxation processes are shown. Electrons from the defect level can directly relax back into the valence band of diamond with a rate $\gamma _{dir}$. Thermal excitation with a rate $\gamma _{thermal}$ can bring electrons from the defect level into the conduction band of $\hbox {Al}_2\hbox {O}_3$, where they relax with a rate $\gamma _{indir}$ back into the valence band of diamond. In both relaxation processes a recombination occurs that decreases the conductivity.

The excitation process that occurs during the illumination is illustrated in Fig. 4a. Electrons from the valence band at the diamond surface are excited into the defect level $E_T$ by photon absorption with the rate $\gamma _{exc}$. The remaining holes are filled by electrons from the Fermi edge, increasing the hole carrier density in the 2DHG and resulting in an increased conductivity (cf. Fig. 2, $t\le 5$ min). Some electrons in these defects relax back into the 2DHG (this is described further below) until a steady state is reached (see Fig. 2, $10\,\hbox {min}\le t\le 60\,\hbox {min}$).

After turning off the laser, the excitation process ceases to occur while the relaxation process still takes place. We expect two different relaxation processes to simultaneously happen, illustrated in Fig. 4b. The first is the direct relaxation from the defect into the 2DHG with the rate $\gamma _{dir}$. Then a recombination with a hole occurs which reduces the carrier density. We assume this relaxation process to be temperature-independent and only dependent on the overlap between the wave functions of the electron in the defect and the hole in the 2DHG.

The second relaxation process is a two-step process. First, electrons in the defects are thermally excited into the conduction band of $\hbox {Al}_2\hbox {O}_3$ with a rate $\gamma _{thermal}$. This rate depends on both temperature and activation energy $\Delta E=E_C-E_T$ and gives rise to the DLTS spectra in Fig. 3. The electron is then trapped at the diamond-$\hbox {Al}_2\hbox {O}_3$ interface. From here, electron-hole recombination takes place with the rate $\gamma _{indir}$ and results in the decreasing hole carrier density.

Based on the discussed mechanisms, it is possible to estimate the defects density. First, it has to be mentioned that the relaxation rates are smaller than the excitation rate, $\gamma _{exc}>\gamma _{thermal}+\gamma _{dir}+\gamma _{indir}$ (see Fig. 2). As a consequence, it can be assumed that nearly all defects are fully charged by the carriers from the valence band of diamond, that lead to the conductivity change in the 2DHG. The (Drude) conductivity of the 2DHG is directly proportional to its carrier density. Therefore, by separating the amplitude of conductivity change by deconvolution of the transients with the time constants from the DLTS evaluation, we are able to estimate the density of each defect. The assumption of negligible relaxation rate leads to a small overestimation of the defect density. The resulting defect densities are listed in Table 1.

While repeating the transient measurement with the infrared laser (850 nm) for sample 2, we could not observe the transients like under UV laser illumination. This verifies our model, as the highest activation energy in sample 2 was determined to be 1.2 eV. This corresponds to an energy of 1.2 eV below the conduction band of $\hbox {Al}_2\hbox {O}_3$. Taking an offset of 3.6 eV between conduction band of $\hbox {Al}_2\hbox {O}_3$ and the Fermi energy of the 2DHG^21^, the energy difference between Fermi energy of the 2DHG and the lowest defect measured in this study is 2.4 eV. This is lower than the 3.06 eV of the UV laser but higher than the 1.46 eV of the infrared laser. On the other hand, it is possible to directly excite charge carriers from the 2DHG into the presented defects using the UV laser. Hence, if the activation energy is not aligned to the conduction band it would be possible to excite electrons from the valence band of diamond into the defects with the infrared laser. In our experiment, this was not observed and therefore strengthens our model.

Defect states in $\hbox {Al}_2\hbox {O}_3$ have mostly been reported by theoretical calculations. The identification of the measured defects is done by comparing the different values of $\Delta E$ with those reported in theoretical studies. Watcharatharapong et al. investigated native point defects in $\hbox {Al}_2\hbox {O}_3$ and found that threefold positively charged aluminum interstitials $\hbox {Al}_{\mathrm{i}}^{3+}$ have an energy level of 0.51 eV below the conduction band^22^. This energy level is close to three defects found in our study, namely defect A, B and D, see Table 1. We conclude that defect A (sample 1) and D (sample 2) are the same defect and that this is $\hbox {Al}_{\mathrm{i}}^{3+}$.

Sankaran et al. studied extrinsic defects in $\hbox {Al}_2\hbox {O}_3$ and found different defect levels for silicon, carbon and nitrogen with $\Delta E$ between 1.6 eV and 2.0 eV^23^. A comparison with the values in Table 1 suggests that defect C in sample 1 ($\Delta E=1.658\,\hbox {eV}$) is silicon-related. We identified a contaminated evaporation boat, used for the $\hbox {Al}_2\hbox {O}_3$ deposition, as a possible source of the Si impurities. To verify this, we replaced the boat before preparation of sample 2. The fact that sample 2 does not show any defect with $\Delta E$ in the range between 1.6 and 2.0 eV strongly supports our conclusion that defect C is related to silicon.

It is difficult to further identify the remaining defect level, as carbon and nitrogen-related defects have a few levels between 0.5 and 1.4 eV^23^, which are equally suited to account for our experimental data.

## Summary and outlook

In summary, we used the 2DHG on hydrogen-terminated diamond as a sensor to determine the activation energies, capture cross sections and densities of five different defect levels in the bandgap of the undoped dielectric material $\hbox {Al}_2\hbox {O}_3$. This shows that our technique is suited for the detection of such defect levels in dielectric materials. Our method demonstrates a high sensitivity, as the total detected charge (i.e. sample area $\times$ surface charge density) was below $10^8$ elementary charges. The technique also proved to be selective. Our method is not limited to slow relaxations. In related experiments on quantum dots, sampling rates higher than 300 MHz have been obtained^24^. The basic principal behind the demonstrated method is very versatile and we suggest to perform this characterization measurements on different dielectric materials in the future and also with different two-dimensional carrier gases. Graphene—as a two-dimensional material which can be transferred onto almost any substrate—would probably be the most promising candidate as a high-sensitive detector for charge defects in dielectric materials.

## Data Availability

The datasets generated during and/or analysed during the current study are available from the corresponding author on reasonable request.
